# Epidemiologic heterogeneity of common mood and anxiety disorders over the lifecourse in the general population: a systematic review

**DOI:** 10.1186/1471-244X-9-31

**Published:** 2009-06-01

**Authors:** Arijit Nandi, John R Beard, Sandro Galea

**Affiliations:** 1 Center for Population and Development Studies, Harvard School of Public Health, Boston, USA; 2 Center for Urban Epidemiologic Studies, New York Academy of Medicine, New York, USA; 3 School of Public Health, University of Sydney, Sydney, Australia; 4 Faculty of Health and Applied Science, Southern Cross University, Lismore, Australia; 5 Department of Epidemiology, University of Michigan School of Public Health, Ann Arbor, USA; 6 Department of Epidemiology, Columbia University Mailman School of Public Health, New York, USA; 7 Survey Research Center, Institute for Social Research, Ann Arbor, USA

## Abstract

**Background:**

Clinical evidence has long suggested there may be heterogeneity in the patterns and predictors of common mood and anxiety disorders; however, epidemiologic studies have generally treated these outcomes as homogenous entities. The objective of this study was to systematically review the epidemiologic evidence for potential patterns of heterogeneity of common mood and anxiety disorders over the lifecourse in the general population.

**Methods:**

We reviewed epidemiologic studies examining heterogeneity in either the nature of symptoms experienced ("symptom syndromes") or in patterns of symptoms over time ("symptom trajectories"). To be included, studies of syndromes were required to identify distinct symptom subtypes, and studies of trajectories were required to identify distinct longitudinal patterns of symptoms in at least three waves of follow-up. Studies based on clinical or patient populations were excluded.

**Results:**

While research in this field is in its infancy, we found growing evidence that, not only can mood and anxiety disorders be differentiated by symptom syndromes and trajectories, but that the factors associated with these disorders may vary between these subtypes. Whether this reflects a causal pathway, where genetic or environmental factors influence the nature of the symptom or trajectory subtype experienced by an individual, or whether individuals with different subtypes differed in their susceptibility to different environmental factors, could not be determined. Few studies addressed issues of comorbidity or transitions in symptoms between common disorders.

**Conclusion:**

Understanding the diversity of these conditions may help us identify preventable factors that are only associated with some subtypes of these common disorders.

## Background

Numerous large epidemiologic surveys have demonstrated the high prevalence of mood and anxiety disorders among the general population. The recent World Health Organization (WHO) World Mental Health Surveys, for example, interviewed 60,463 community-based adults living in 14 countries. These studies estimated the 12 month prevalence of mood disorders in developed countries at between 3.1 percent in Japan and 9.6 percent in the US, and the prevalence of anxiety disorders at between 5.3 percent and 18.2 percent [1]. The US National Comorbidity Replication found lifetime prevalence estimates for these conditions of 20.8 percent for mood disorders and 28.8 percent for anxiety disorders [2]. These high prevalence estimates are associated with a heavy burden on the health of the community, with most individuals categorized with a disorder having clinically significant symptoms and suffering a significant associated disruption to their daily life [3]. According to 2004 estimates from the WHO, neuropsychiatric disorders are the leading cause of disability among non-communicable conditions worldwide [4].

Clinical experience has long suggested that mood and anxiety disorders are heterogeneous syndromes that vary markedly between individuals with respect to their clinical presentations, responses, longitudinal course, and risks of recurrence. According to Thase (2007), for example, the origins of the atypical depressive subtype can be traced back to the work of Sir Aubrey Lewis, who in the 1930s proposed dividing depression into endogenous or nonendogenous subtypes, a partition supported by the psychopharmacologic work of West and Dally in 1959 [5]. In 1982, Sheehan and Sheehan similarly proposed an alternative classification scheme for phobic disorders based on the presence or absence of endogenous anxiety symptoms; the two subgroups differed with respect to their clinical presentation, response to treatment, and longitudinal course [6]. Prospective work has shown that patients with mood and anxiety disorders follow different longitudinal trajectories that vary in terms of age or onset, symptom severity, and risks of recurrence [7-11]. For example, in a prospective study of 120 patients treated for current major depressive disorder, Ceroni and colleagues (1984) found that the majority of patients recovered within the first few months of treatment, but 39 percent were persistently depressed during the first year of follow-up [8]. Additionally, in a prospective study of 83 moderate to severely depressed patients, 20 percent were almost entirely free of depressive symptoms over ten years of follow-up, while 5 percent were continuously depressed [7].

The modern paradigm for the diagnosis of mental disorders is based on the classification systems of the DSM and ICD. Accordingly, most recent population-based research has used different survey instruments to define the presence of mood and anxiety disorders based on these criteria, either in the form of a categorical diagnosis or as symptom severity levels on a unidimensional scale. These approaches have greatly increased our understanding of these disorders and identified a range of risk factors for new onsets [12-14]. However, failing to account for heterogeneity in the clinical presentations of mood and anxiety disorders comes at a cost. If, for example, a specific risk factor was only associated with a particular subtype of a disorder, this may be overlooked in an analysis investigating all mood disorders as the outcome; there is some evidence to suggest this may be the case [15]. Incomplete understanding of the specific etiologic pathways that manifest in distinct phenotypes has important implications for the translation of research into effective treatment and clinical management [16]. This is only one of several criticisms levied against current models of classification in a growing appeal for a new taxonomy that appreciates the heterogeneous nature of mood and anxiety disorders highlighted by earlier clinical work [17-19].

**Figure 1:**
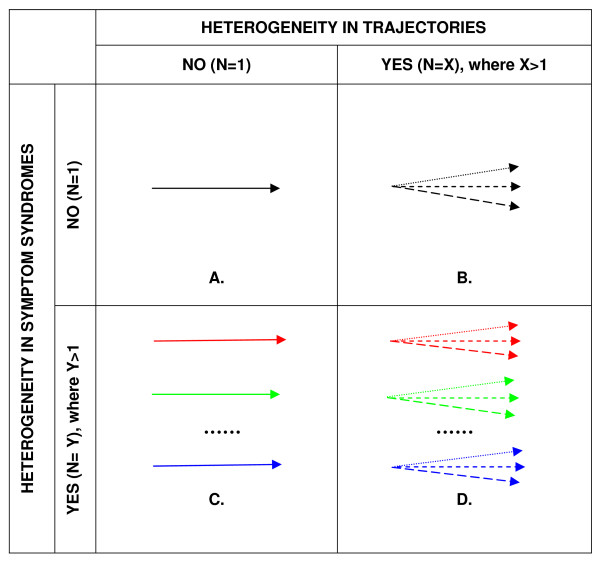
**Potential patterns of heterogeneity in symptom syndromes and trajectories of common mood and anxiety disorders**. **Notes**: A. represents homogeneity; B. represents the X potential phenotypes resulting from heterogeneity of trajectories but not symptom syndromes; C. represents the Y potential phenotypes resulting from heterogeneity of symptom syndromes but not trajectories; D. represents the X*Y potential phenotypes resulting from heterogeneity of both symptom syndromes and trajectories.

Facilitated by methods such as latent class analysis, a growing body of epidemiologic research has attempted to disentangle phenotypic heterogeneity of common mood and anxiety disorders by identifying clusters of symptoms. Consistent with extant clinical and population-based research, we propose that potential patterns of heterogeneity can be categorized as relating to clusters of symptoms, according to clinical features or severity ("symptom syndromes"), and patterns of symptoms over time ("symptom trajectories"). Figure 1 summarizes the potential patterns of heterogeneity of symptom syndromes and trajectories of common mood and anxiety disorders observed. It is the goal of this paper to systematically review the epidemiologic evidence for potential patterns of heterogeneity in both the symptom syndromes and in the trajectories of common mood and anxiety disorders. We restricted our review to population-based studies, as studies of the life course of these disorders need to include the large number of individuals with significant symptoms who do not seek appropriate clinical care and who would be excluded from studies drawn only from clinical populations [20]. It is hoped that this review will be useful in pointing the way to further research and potentially to more effective intervention strategies.

## Methods

### Selection criteria

The sampling frame for this review included population-based studies that assessed the heterogeneity of symptom syndromes or trajectories of common mood and anxiety disorders. We restricted our review to psychiatric definitions of common mood and anxiety disorders and, as such, these disorders were selected based on the taxonomy of the DSM-IV [21]. We also based our review on DSM-IV definitions of mood and anxiety disorders because most studies assessing heterogeneity in these conditions appeared in the peer-reviewed literature after 1994, the year the DSM-IV was published, and also because we wanted to minimize the extent to which heterogeneity in the symptom syndromes or trajectories of common mood and anxiety disorders was an artifact of changing nosology over time. Studies of the heterogeneity of symptom syndromes were required to identify distinct symptom subtypes. Studies of the heterogeneity of trajectories were required to identify distinct longitudinal patterns of symptoms, or the characteristics of these trajectories, in at least three waves of follow-up. Studies based on samples recruited from clinical settings (e.g., inpatients or outpatients) were excluded.

### Search strategy

We obtained papers for this review using a four-step procedure. First, because our review was based on DSM-IV definitions of the mood and anxiety disorders, we performed a systematic search of the peer-reviewed literature using the Index Medicus and ISI Web of Knowledge databases. We identified potential studies for inclusion by querying all possible search fields for combinations of the following terms: 'anxiety', 'mood', 'disorder', 'heterogeneity', ' symptom', 'subtype', 'symptom subtype', 'trajectory', 'trajectories', 'depression', 'posttraumatic stress', 'PTSD', 'obsessive', 'compulsive', and 'ADHD'. Second, we analyzed abstracts for all studies identified and excluded papers that did not satisfy selection criteria. Third, we analyzed the full-text version of all remaining studies and excluded those that did not satisfy selection criteria. Fourth, we retrieved articles not identified by our literature review from the references of remaining papers and excluded those that did not satisfy selection criteria.

### Search results

Our search identified 521 papers, 46 of which satisfied selection criteria. In Table one and Table two (Additional files 1 and 2), we present all findings within two broad categories according to whether they assessed heterogeneity of symptom syndromes (n = 17) or heterogeneity of trajectories (n = 29), respectively. Within each table, studies were further stratified based on the particular mood and anxiety disorder assessed (e.g., depression, posttraumatic stress disorder) and then sorted in ascending order based on the age group of the sample (i.e., adolescent, adult, elderly) and alphabetically based on the first author's last name. Each table provides a summary of the sample, the sample size, the authors, and the study design in the first column, the age group of the sample in the second column, the timeframe of interviews in the third column, and the main findings in the fourth column. These tables aim to highlight the most meaningful conclusions from the studies collected.

## Results

### Heterogeneity in symptoms of mood and anxiety disorders

We identified 17 studies that evaluated heterogeneity in symptoms of mood and anxiety disorders (Additional file 1). Of the 17 studies, 10 studies assessed depression, three studies assessed social phobia, and there was one study each on PTSD, ADHD, bipolar disorder, and panic attack. One study focused on adolescents, 15 on adult samples, and one on elderly adults. Fifteen of 17 studies were cross-sectional. Seven of 17 studies distinguished clinical subtypes of bipolar disorder, depression, and panic attack based on definitions specified *a priori*. Ten studies relied on different statistical methods, most frequently latent class analysis, to identify subtypes based on observed data.

#### Evidence for distinct symptom subtypes

Eleven of the 17 studies focused on the identification of distinct clinical subtypes of mood and anxiety disorders. For depression, two general population samples [22,23] and two samples of twins from population-based registries [24,25] separated adults into latent classes based on their symptom patterns. In their general population sample of adults from Baltimore, Chen and colleagues (2000) identified five latent classes with distinct patterns of depressive symptoms, including non-depressed, anhedonic, suicidal, psychomotor, and severely depressed subtypes [23]. Using latent class analysis, Sullivan and colleagues (1998) identified six patterns of depressive symptoms in a probability sample of the US population, including a severe typical subtype with a high lifetime occurrence of depressive symptoms, a severe atypical subtype with many depressive symptoms and symptoms characterized by appetite increase and weight gain, and four other subtypes of varying symptom severity [22]. Similarly, two twins studies using latent class analysis, including a study of female-female twin pairs [25] and a study of male-male and male-female twin pairs [24], identified a severe typical depressive subtype where nearly all criteria for major depression were met, an atypical depressive subtype where participants commonly endorsed symptoms of depressed mood, loss of interest and/or pleasure, and increased appetite and weight gain, and five additional subtypes varying according to their patterns and degree of classical depressive symptoms. In contrast to these results demonstrating subtypes of symptom syndromes of depressive disorders, a study of elderly adults found little evidence for a clinically defined vascular subtype of depression in the general populations of Amsterdam and Rotterdam [26].

Four studies attempted to identify subtypes of social phobia [27-30]. In a general population sample of adults from Sweden, Furmark and colleagues (2000) found evidence of severe, intermediate, and mild subtypes that were distinguished by the symptom severity of their social phobia [27]. In contrast, a study using principal components analysis found that the number of social fears in their sample of young German women was distributed continuously with no clear evidence for distinct symptom subtypes based on the number of symptoms of social phobia [30]. In a latent class analysis that assessed clusters of symptoms patterns, Kessler and colleagues (1998) identified a subtype that endorsed few symptoms of social phobia, a subtype characterized by fears related to social speaking, and a subtype with multiple speaking and non-speaking fears in the general population of the US [28]. Two other studies provided evidence that those with social phobia characterized by only speaking-related fears may represent a distinct subtype from those with non-speaking social fears [28-30].

There was one study each on posttraumatic stress disorder (PTSD) and attention-deficit/hyperactivity disorder (ADHD). Using a taxometric analysis, Waelde and colleagues (2005) found evidence of a dissociative subtype of PTSD among Vietnam theater era veterans that was characterized by a higher prevalence of symptoms of dissociation, PTSD, and dysthymia [31]. In a latent class analysis of adolescent female twins aged 13 to 23 years, three ADHD subtypes of clinical interest, among nine total subtypes, were identified, including an inattentive subtype without comorbidity, an inattentive subtype with increased symptoms of oppositional defiant disorder (ODD), and a combined inattentive/hyperactive-impulsive subtype with elevated levels of ODD, separation anxiety and depressive symptoms [32].

#### Factors associated with symptom subtypes

Thirteen studies assessed whether specific characteristics might be associated with subtypes of common mood and anxiety disorders. Two studies comparing the class assignment of twins showed that monozygotic twins were more likely to be assigned to the same latent subtypes of depression and ADHD than dizygotic twins [24,32], suggesting a potential genetic influence on class membership. Two general population samples showed that personal and familial characteristics discriminated between latently defined subtypes of depression [22,23]. For example, a study of Baltimore adults showed that a family history of depression was associated with membership in the anhedonic, psychomotor, suicidal, and severely depressed subtypes, female gender was associated with the suicidal and several depressed subtypes, and exposure to stressful life events was associated with psychomotor and suicidal subtypes [23]. Sociodemographic factors, including levels of income, educational attainment, and social support also distinguished between subtypes of social phobia. In general, subtypes characterized by greater symptom severity or functional impairment, including those with both speaking and non-speaking social fears relative to those with only speaking fears [30], were associated with lower levels of income, education, and social support than milder subtypes [27,28]. A number of studies showed that comorbid anxiety, mood, and substance disorders were more common among some depressive [22,33] and panic [34] subtypes than others. For example, one study showed the atypical major depression subtype was associated with an increased prevalence of comorbid panic disorder and drug abuse/dependence [33], whereas a national sample of US adults showed that more deviant personality and attitudes, increased psychiatric comorbidity, and parental alcohol/drug use were associated with membership in severe typical or atypical depressive classes relative to milder subtypes [22]. Three studies estimated the prevalence of diagnostically defined seasonal subtypes of depression and bipolar disorder [35-37]; two of these studies investigated the influence of environmental factors, but found no association between the latitude of a participants' household dwelling in Ontario and the prevalence of the seasonal subtypes of depression [36] or bipolar disorder [35]. A few studies compared the course of depressive subtypes. For example, one study of young adults from Zurich showed that the course of diagnostically defined atypical depression was associated with an earlier age of onset and greater chronicity [38].

### Heterogeneity in the trajectories of mood and anxiety disorders

Of the 29 studies that assessed heterogeneity in the trajectories of mood and anxiety disorders (Additional file 2), 22 assessed depression, three assessed anxiety, two assessed general anxiety and depression, one assessed symptoms of hyperactivity, and one assessed PTSD. Fifteen studies focused on children, adolescents, or young adults, eight on adults, five on elderly adults, and one on a sample of mixed age groups. Studies applied different statistical techniques, most commonly latent growth curve or semi-parametric group-based modeling, to identify trajectories of mood and anxiety disorders on the basis of the observed data.

#### Evidence for distinct trajectories

Six studies identified distinct longitudinal trajectories of depressive symptoms among children, adolescents, or young adults [39-45]. For example, in a semi-parametric group-based analysis of early adolescents from Northwestern Quebec, Brendgen and colleagues (2005) identified consistently low, moderate, increasing, and consistently high trajectories of depression; almost 50 percent of participants were in the consistently low group [39]. Additionally, a study of African American adolescents from a mid-Western city who were at risk of high school dropout identified consistently high, consistently low, increasing, and decreasing depressive trajectories [41]. Most recently, Costello and colleagues (2008) identified four trajectories of depression in a nationally representative sample of 12 to 25 years olds; 29 percent were assigned to the group without depressed mood, 59 percent were assigned to the stable low depressed mood, 10 percent were assigned to the declining depressed mood group, and two percent were assigned to the late escalating depressed mood group [45]. Three studies assessed depressive trajectories among adults, including two studies of caregivers [46-48]. Using a semi-parametric group-based analysis, Campbell and colleagues (2007) identified low-stable, moderate-stable, intermittent, moderate-increasing, high-decreasing, and high-chronic patterns of depressive symptoms among mothers as their children aged from one month to seven years, with more than 80 percent of mothers in the low-stable or moderate-stable trajectory groups [46]. A latent state-trait analysis of elderly residents from the Baltimore area found that heterogeneity in depressive symptoms was accounted for by two factors, a highly heritable trait effect that reflects underlying vulnerability and a residual state effect that reflects occasion specific circumstances [49]. Six trajectory groups, including two asymptomatic groups, a stable low-depressed group, an emerging depressive symptoms group, a remitting depressive symptoms group, and a persisting depressive symptoms group, were identified in a community sample of elderly adults from rural southwestern Pennsylvania [50].

Two studies used semi-parametric group-based models to identify distinct trajectories of symptoms of anxiety among children. In a representative sample of children from Quebec, Duchesne and colleagues (2008) identified low, moderate, high, and chronic trajectories of symptoms of anxiety [51]. A study of boys enrolled in the WIC program in Pittsburgh also identified four trajectories of symptoms of anxiety, including low, low-increasing, high-declining, and high-increasing groups [52]. In contrast to the Quebec study, where 40 percent of the sample was assigned to the high severity group, 50% of boys from Pittsburgh were assigned to the low anxiety trajectory. Additionally, two studies examined heterogeneity in the trajectories of both mood and anxiety disorders. A sample of 4,627 members of the 1946 British birth cohort were followed from age 13 through 53, with 44.8 percent considered to have no symptoms, 33.6 percent having repeated minor or moderate symptoms generally below threshold of mental illness, 11.3 percent having few symptoms in adolescence but minor or moderate symptoms in adulthood, 5.8 percent having symptoms in adolescence but not in adulthood, 2.9 percent having few symptoms in adolescence but severe symptomatology in adulthood, and 1.7 percent having persistent or repeated severe symptoms [53]. In a representative sample of adults from Zurich, Merikangas and colleagues (2003) used log-linear models to investigate the relation between anxiety, depression, and comorbid anxiety and depression. This study showed that comorbid anxiety and depression was more stable over time than either anxiety or depression alone and that transitions from anxiety only to depression only were common, whereas transitions from depression only to anxiety only were rare [54].

One study used semi-parametric group-based models to assess heterogeneity in the trajectories of hyperactivity symptoms; in that study, four trajectories of symptoms, including very low, low, moderate, and high groups, were identified in a nationally representative sample of Canadian children [55]. With regards to PTSD, a study of Army personnel who returned from the Gulf War used growth mixture modeling to identify two trajectories of post traumatic stress disorder; 57 percent of participants were assigned to the first group, which had lower post traumatic stress disorder symptomatology at baseline and showed slight increases over time, and 43 percent of participants were assigned to the second group, which had higher levels of initial symptoms and a significant increase over time [56].

#### Factors associated with distinct trajectories

17 studies assessed the correlates of distinct depressive trajectories among children, adolescents, and young adults. Trajectories of depressive symptoms [39-41,44,57-60] and symptoms of anxiety [61] have been frequently shown to differ by gender. For example, some studies showed that female gender was associated with membership in more severely depressed groups [39,41]. Other work showed that girls' depressive symptoms increased through early and middle adolescence while boys' symptoms remained relatively constant [57,58]; differential exposure to stressful life events may explain observed gender differences in depressive trajectories [58].

Besides demographic factors such as age, family characteristics, emotional and personality traits, sociodemographic factors, performance in school, substance abuse and other comorbidities, and exposure to stress, stressful life events, and negative life events were commonly associated with adverse symptom trajectories of anxiety, depression, and hyperactivity in children and adolescents [41-45,52,55,57-59,61]. Characteristics of parents, including maternal prenatal smoking, maternal depression, and hostile parenting practices, were associated with more symptomatic trajectories of anxiety and hyperactivity among children and adolescents [52,55]. Sociodemographic factors, including non-white race/ethnicity and lower socioeconomic status were associated with membership in depressed mood trajectory groups relative to groups without symptoms of depression [45]. Several studies showed that poorer performance in school was associated with greater severity of depressive symptoms [41,42,59]; for example, a study of African American adolescents from a mid-Western city found that adolescents who presented with consistently high levels of depressive symptoms were more likely to have lower grade point averages compared with adolescents in other groups [41]. Comorbidities, including adolescents' smoking, alcohol consumption, and illicit drug use [42,45,60], poorer social relationships among adolescents, particularly with their parents and same sex peers [39,59], and greater parental educational attainment have also been associated with more adverse adolescent depressive symptoms trajectories [59]. Conversely, social supports and marriage were associated with lower levels of depressive symptoms among young adults in a Western Canadian city [59].

Six studies assessed the determinants of distinct depressive trajectories among adults, with many examining the influence of socioeconomic circumstances. In a study of Southeast Asian refugees in Vancouver more economically integrated refugees showed higher initial levels of subclinical depressive symptomatology, but greater declines over time [62]. In a study of mothers followed from one month to seven years after the birth of their child, greater educational attainment and a higher income to needs ratio, among other factors, were associated with low-stable levels of depression relative to more severe depressive trajectories [46]. Similarly, Li (2005) found that while wife and daughter caregivers with higher incomes were more likely to exhibit a downward trajectory of depressive symptoms that began before their care recipients died, caregivers with lower incomes and caregivers of recipients with more problematic behaviors were slower to recover after recipients died [63]. In contrast to these results, a US national study of 3,617 adults did not find a difference in trajectory between high and low income groups, although there was divergence over time between college graduates and those with less than a high school diploma [64].

The most commonly studied determinant of depressive trajectories among the elderly has been stress. Using a probability sample of 1,972 Black and White adults age 65 and older from five counties in North Carolina, a significant association was found between stress growth and growth of depressive symptoms, particularly among Blacks [65,66]. On the other hand, a study of community-dwelling adults aged 65 and older from areas of North Carolina showed that the positive relation between age and depressive symptoms was driven primarily by differences between cohorts, and that adjustment for indicators of the life course (i.e., marriage, socioeconomic status, employment status), physiological declines, and sex compositions largely explained these cohort effects [67].

Only single studies have examined determinants in the trajectories of either PTSD or mood and anxiety disorders combined among adults. For PTSD, White Army personnel returning from the Gulf War and those with higher educational attainment and less combat exposure had a lower likelihood of reporting high levels of posttraumatic stress symptoms [56]. Among the participants from the 1946 British birth cohort, lower birthweight, older age at first standing, female gender, and manual social class were associated with more severe trajectories of anxiety and depressive symptoms [53].

## Discussion

While only a limited amount of work has been conducted in this field, we found growing epidemiologic evidence that, not only can mood and anxiety disorders be differentiated by symptom syndromes and trajectories, but that the factors associated with these disorders may vary between these subtypes.

Most population-based epidemiologic research investigating heterogeneity in the symptom syndromes of common mood and anxiety disorders has focused on major depressive disorder, with only sparse work relating to ADHD, bipolar disorder, panic attack, PTSD, or social phobia. This work suggests that depression may not be a homogenous disorder characterized by a single phenotype (Figure 1A), but a heterogeneous constellation of potentially distinct subtypes of symptom syndromes (Figure 1C). For depression, the syndromic subtype most commonly identified in this review was atypical depression, a subtype defined by symptoms including increased appetite and weight gain and greater chronicity. Although the validity of atypical depression has been debated since the subtype was codified in the DSM-IV in 1994 [5,68,69], three latent class analyses provided support for an atypical subtype that was distinct in terms of its patterns and severity of symptoms [22,24,25]. However, these analyses did not support the presence of mood reactivity as a necessary symptom for the diagnosis of atypical depression, a finding corroborated by recent clinical work [69]. There was less evidence for other clinical subtypes of depression, including melancholic, seasonal, and vascular depression [26,36,37]. For example, an anhedonic latent class exhibiting a greater loss of interest was identified by one study [23]; however, other work showed that these symptoms were non-specific [24]. Additional epidemiologic replication of these clinical subtypes is needed. Additionally, although cross-sectional and longitudinal research has identified high rates of comorbidity of mood and anxiety disorders, few studies of symptom syndromes explored symptoms of more than one disorder. These are some of the limitations that will have to be addressed for a new typology to emerge.

Familial aggregation studies suggest that heterogeneity in the symptom syndromes of depression and ADHD are partly explained by genetic similarities [24,32]. However, the particular genetic, sociodemographic, psychological, social, or environmental characteristics that explain observed heterogeneity in symptoms is unclear; many of these characteristics may lie along the same etiologic pathway. Future research will have to address a number of issues. First, because most studies were cross-sectional and started in adulthood, it was impossible to distinguish associations that may reflect a causal pathway from those that may be spurious. Longitudinal assessments that establish a temporal structure between risk factor and phenotype will help to understand observed associations. Second, most of the associations between exposures and subtypes were non-specific in nature. For example, analysis of the National Comorbidity Survey showed that depressive atypicality, a subtype based on patterns of symptoms, was associated with interpersonal dependency, reduced self esteem and stressful life events [22,24]. Similarly, analysis of symptom subtypes in the Baltimore Epidemiologic Catchment Area study found more severe subtypes were associated with female gender and family history but not stressful life events, while mild or moderate cases were associated with family history and stressful events, but not female gender [23]. Although it is plausible that one exposure may be associated with multiple subtypes, further phenotypic characterization of these disorders will increasingly help disaggregate these relations and facilitate identification of the specific genetic, personal, and social factors associated with distinct symptom subtypes [16].

We found strong evidence of heterogeneity in the longitudinal trajectories of common mood and anxiety and disorders. There are three potential explanations for these patterns. First, heterogeneity in trajectories may be the result of having distinct symptom subtypes in the population, each of which may have a distinct longitudinal course (Figure 1C). In this case, heterogeneity in symptom syndromes may be spuriously confused as heterogeneity in the longitudinal trajectories of mood and anxiety disorders. Second, there may be true intra-individual heterogeneity in the longitudinal course of a single clinical disorder because of differential exposure to genetic, personal, or environmental factors. Therefore, a homogenous clinical disorder may present as multiple phenotypes (Figure 1B). Third, there may be intra-individual heterogeneity in the longitudinal course of multiple symptom subtypes (Figure 1D). Overall, our review found stronger evidence for intra-individual heterogeneity in the longitudinal course of a single disorder than heterogeneity resulting from having distinct clinical subtypes in the population. Several studies conducted among children and adolescents found evidence of distinct depressive trajectories characterized by different levels of symptom severity. In general, the most prevalent trajectories were stable patterns of consistently low to moderate levels of symptoms [39,40,42,45]; however, up to one-quarter of some samples were assigned to classes characterized by persistent levels of severe symptomatology [42,43].

Only sparse research has investigated intra-individual heterogeneity in the longitudinal course of multiple symptom subtypes. In that study, Angst and colleagues (2002) found that atypical depression was characterized by greater longitudinal chronicity [38]. Studies that examined trajectories of mood and anxiety disorders combined were also very limited, although both clinical and epidemiologic evidence suggests that may individuals experience frequent transitions between symptoms of these disorders over time. It is possible that individuals who experience symptoms of both disorders over a lifetime share characteristics that distinguish them from individuals who only experience symptoms of one specific disorder. This seems an area worthy of further investigation.

There was strong evidence that a variety of factors experienced over the lifecourse, ranging from personal to social, may influence trajectories of common mood and anxiety disorders. There was also evidence to suggest that vulnerability to external factors may vary between individuals with differing trajectories. Extended longitudinal studies starting in childhood are needed to distinguish between these effects. The extant literature suggests that certain characteristics may predispose individuals to membership in more severe, versus less severe, longitudinal trajectories of depression. For example, among children, adolescents, and young adults, female gender, poorer school performance, greater exposure to stressful life events, and comorbidities including substance use were all associated with trajectories characterized by greater depressive symptomatology [41-44,57-60]. Similarly, among adults, increased demands among caregivers were associated with more severe depressive trajectories [63]. Conversely, a number of studies suggest that certain factors, particularly greater access to social and material resources, may act as buffers and predispose individuals to less severe trajectories of depression. Among adolescents and young adults, research showed that stronger social relationships and greater access to social supports predicted membership in trajectories characterized by less severe symptoms of depression [39,59]. Furthermore, one study showed that greater parental educational attainment was associated with a steeper decline in adolescents' depressive symptoms [59], suggesting that socioeconomic status may be associated with less severe depressive trajectories. Among adults, greater educational attainment and financial resources were associated with less severe depressive trajectories [46,63]. As with symptom syndromes, whether environmental factors are the cause of a particular trajectory, or whether individuals with a genetic predisposition to a particular trajectory are more susceptible to specific environmental factors, can only be answered by extended longitudinal studies and the assessment of interaction between genetic and environmental factors [70,71]

Finally, an alternative way of viewing these patterns was proposed by twin studies and the Baltimore Longitudinal Study of Aging, which both support a trait-state model of depression, where symptom levels can be accounted for by two factors: a level (average or "trait") effect that is highly heritable and reflects underlying vulnerability and a residual ("state") effect that is non-inheritable and reflects occasion specific circumstances [49,71]. In this theoretical framework, trajectory subtype may be considered a manifestation of trait effects. These studies concluded that attempts to identify environmental determinants of symptoms of depression might best focus on *deviations *about average levels over multiple assessments.

### Methodological challenges

Further inference about the heterogeneity in symptom subtypes and trajectories of common mood and anxiety disorders over the lifecourse is limited by a number of methodological issues. First, the studies included in this review were population-based and utilized a variety of diagnostic survey instruments that are, to varying degrees, imperfect substitutes for clinician-administered structured interviews. Error in the measurement of the mood and anxiety disorders may influence the validity of individual studies and complicate comparisons between studies. Further information on the validity of common diagnostic instruments can be found elsewhere [72]. Second, in most studies that assessed the characteristics associated with distinct clinical presentations or trajectories, methods of variable selection were not theoretically predicated. This makes it difficult to assess whether a particular characteristic was associated with heterogeneity in mood and anxiety disorders across studies. A multilevel framework for understanding how factors experienced over the life course influence the heterogeneity of common mood and anxiety disorders may facilitate model specification and improve comparability between studies. Third, studies used a number of different methods for defining subtypes. Studies that assessed heterogeneity of clinical presentations either specified criteria *a priori or *used statistical methods to identify subtypes. In general, disorders recognized as distinct clinical entities by the DSM-IV, including seasonal and atypical depression, were more likely to rely on definitions specified *a priori *than less commonly studied subtypes. The dichotomy between pre-defined and empirically derived subtypes represents a bias-variance trade-off. While having pre-defined criteria facilitates comparisons between studies, there is ongoing debate about the validity of recognized subtypes [73,74]. On the other hand, methods such as latent class analysis are more flexible and permit investigation of the number and nature of underlying subgroups in a sample [75,76]. However, these techniques can be overly sensitive to the data and may complicate comparisons across studies. For example, are the mild depressive classes from two different studies qualitatively similar [22,25]? This trade-off was not relevant when considering studies assessing heterogeneity in trajectories of mood and anxiety disorders. These studies typically used latent class growth, semi-parametric group-based modeling, or other techniques to identify distinct trajectories according to the inferential goal of the analysis [77,78]. Fourth, despite the clinical and epidemiologic evidence for frequent comorbidity and transitions between mood and anxiety disorders, more than 90 percent of the studies identified by our review assessed symptoms of depression alone. Comorbidity with, and transitions between, these disorders is likely to be a field particularly worthy of further investigation. Finally, most studies did not distinguish between age and cohort effects.

## Conclusion

This is the first review to explore the epidemiologic evidence for heterogeneity of mood and anxiety disorders. Clinical experience suggests these common conditions vary markedly between individuals, and the limited epidemiologic studies conducted in this area are consistent with these observations. This is important since this research also suggests that the factors associated with these disorders may vary by symptom and trajectory subtype. These associations may be overlooked in epidemiologic studies that consider these outcomes as homogeneous entities. Understanding the diversity of these conditions may help us identify preventable factors that are only associated with some subtypes of these common disorders. This knowledge may aid the development of more effective treatment interventions.

## Abbreviations

DSM-IV: Diagnostic and Statistical Manual of Mental Disorders, Fourth Edition; PTSD: posttraumatic stress disorder; ADHD: attention-deficit/hyperactivity disorder; ODD: oppositional defiant disorder

## Competing interests

The authors declare that they have no competing interests.

## Authors' contributions

All authors contributed equally to the conception of the study, the interpretation of results, and the drafting of the manuscript. AN was responsible for the acquisition of data. All authors read and approved the final manuscript.

## Pre-publication history

The pre-publication history for this paper can be accessed here:



## Supplementary Material

Additional file 1 **Table one**. Key studies assessing heterogeneity of symptom syndromes of common mood and anxiety disorders. Click here for file

Additional file 2 **Table two**. Key studies assessing heterogeneity of trajectories of common mood and anxiety disorders. Click here for file
