# Supplementary material for: Epidemiologic heterogeneity of common mood and anxiety disorders over the lifecourse in the general population: a systematic review
Source: BMC Psychiatry. 2009 Jun 1;9:31. doi: 10.1186/1471-244X-9-31 (PMC2700109; doi:10.1186/1471-244X-9-31)
Supplement: Additional file 1 — Table one. Key studies assessing heterogeneity of symptom syndromes of common mood and anxiety disorders. [file 1471-244X-9-31-S1.docx]

**Table 1**

Key studies assessing heterogeneity of symptom syndromes of common mood and anxiety disorders

| **SAMPLE; N; [REF#]** | **AGE GROUP** | **METHODS** | **MAIN FINDINGS** |
| --- | --- | --- | --- |
| ***Attention-deficit/hyperactivity disorder (ADHD)*** | | | |
| Adolescent female twins aged 13 to 23 years from a database of all births to Missouri-resident parents for the years 1968-1996; N=2904; [32] | Child/ Adolescent/ Young adult | - Timeframe: Ongoing interviews - Measures: Parents’ responses to 18 ADHD, 8 ODD, and 8 separation anxiety symptoms from the DSM-IV - Analysis: Latent class analysis | *Identification of subgroups:*   - The 9 distinct latent classes identified were: Class 1 (36.3%) with few symptoms; Class 2 (11.2%), described as a depressed group; Class 3 (11.2%), which endorsed symptoms of oppositional defiant disorder (ODD); Class 4 (10.1%), which could not be characterized in terms of any ADHD symptom category; Class 5 (8.9%), described as an ADHD inattentive group; Class 6 (6.8%), which endorsed the most symptoms of separation anxiety; Class 7 (5.7%), which endorsed symptoms of ODD, separation anxiety, and depression; Class 8 (5.3%), with heightened levels of ADHD inattentive and ODD; and Class 9 (4.6%), which included twins with greater inattentive and hyperactivity-impulsive items - Overall, 66.1% of the MZ twins were assigned to the same latent class contrasted with 36.4% of the DZ twins, consistent with a genetic hypothesis for latent class membership |
| ***Bipolar disorder*** | | | |
| Population-based sample of residents of household dwellings in Ontario; N=1605; [35] | Adult | - Timeframe: Interviews in 1996-7 - Measures: Seasonal bipolar disorder assessed using DSI-Ontario Version, based on DSM-III criteria - Analysis: Pre-defined subtypes | *Occurrence of subgroups:*   - Among 62 participants identified as having bipolar disorder, 14 (22.6%) had the seasonal subtype   *Characteristics of subgroups:*   - The prevalence of the seasonal subtype was not associated with latitude |
| ***Depression*** | | | |
| Population-based sample of young adults representative of the canton of Zurich in Switzerland; N=591; [38] | Adult | - Timeframe: Baseline interview in 1978 at ages 19-20 with follow-ups in 1979, 1981, 1986, 1988, and 1993 - Measures: SCL-90-R - Analysis: Pre-defined subtypes based on DSM-IV | *Occurrence of subgroups:*   - According to DSM-IV criteria, the prevalence of the atypical major depressive episodes in the community was 4.0% (4.5% among women and 1.2% among men)   *Characteristics of subgroups:*   - The course of atypical depression was characterized by an earlier age of onset, greater number of days with depressive symptoms, and greater chronicity |
| General population sample of adults from Baltimore; N=1768; [23] | Adult | - Timeframe: Baseline interview in 1980-3 with follow-up in 1993-6 - Measures: DIS - Analysis: Latent class analysis | *Identification of classes based on the number of depressive symptom groups:*   - Being in the mild and moderate groups (3-4 symptom groups) was associated with family history of depressive disorders and stressful life events and being in the severe group (7-9 symptom groups) was associated with family history and female gender, but not stressful life events   *Identification of classes based on latent symptom patterns:*   - The 5 distinct latent classes identified were: non-depressed (81%), with low probabilities of all symptoms; anhedonia (1.8%), with a higher probability of loss of interest, but were not likely to report dysphoria or suicidal thoughts; suicidal (8.3%), with a high probability of dysphoria and and were more likely to report suicidal thoughts/attempts and feeling worthless; psychomotor (5.9%), with a high probability of dysphoria and slowness or restlessness; and severely depressed (3.0%), with high probabilities for all symptoms   *Characteristics of subgroups:*   - The course characteristics were similar among all classes except for the anhedonia class, which showed a older age of onset and fewer lifetime episodes than the other classes - The anhedonia class was associated only with a family history of depression, the psychomotor class was associated with family history and stressful life events, the suicidal class was associated with family history, stressful events, and female gender, and the severely depressed class was associated with family history and female gender but not stressful life events |
| General population sample of adults with typical or atypical major depression from New Haven, Baltimore, St. Louis, Durham, and Los Angeles; N=662; [33] | Adult | - Timeframe: Not reported - Measures: DIS - Analysis: Pre-defined subtypes | *Occurrence of subgroups:*   - The crude prevalence of major depression with atypical features among those with major depression was 16%   *Characteristics of subgroups:*   - Those with atypical major depression were more likely than those with typical major depression to report younger age of onset of major depression and comorbid panic disorder and drug abuse/dependence |
| Adult female-female monozygotic and dizygotic twin pairs from a population-based registry; N=1029; [25] | Adult | - Timeframe: Not reported - Measures: 14 DSM-III-R symptoms of major depression - Analysis: Latent class analysis | *Identification of subgroups:*   - The 7 distinct latent classes identified were: Class 1, in which two-thirds denied any symptoms and one-third endorsed one symptom; Class 2, in which all reported increased appetite and none met criteria for major depression; Class 3, where the most commonly endorsed symptoms were the core symptoms of depressed mood or loss of interest and/or pleasure; Class 4, in which most endorsed decreased appetite and depressed mood; Class 5 (mild typical depression), where nearly all reported depressed mood and more than half described loss of interest and/or pleasure, decreased appetite, trouble sleeping, feelings of tiredness, and psychomotor agitation; Class 6 (atypical depression), where participants commonly endorsed depressed mood, loss of interest and/or pleasure, and increased appetite and weight gain; and Class 7 (severe typical depression), where nearly all met criteria for major depression   *Characteristics of subgroups:*   - In contrast to Classes 5 and 7, Class 6 was characterized by increases in the vegetative functions of eating and sleeping |
| Population-based sample of residents of household dwellings in Ontario; N=1605; [36] | Adult | - Timeframe: Interviews in 1996-7 - Measures: Seasonal depression assessed using DSI-Ontario Version, based on DSM-IV criteria - Analysis: Pre-defined subtypes | *Occurrence of subgroups:*   - The crude prevalence of lifetime seasonal depression was 2.6%   *Characteristics of subgroups:*   - There was no impact of latitude on the prevalence of either major depression or the seasonal subtype |
| Population-based sample of residents of Toronto; N=781; [37] | Adult | - Timeframe: Interviews in 1992-3 - Measures: Seasonal depression assessed using DSI-Toronto Version, based on DSM-III criteria - Analysis: Pre-defined subtypes | *Occurrence of subgroups*   - The prevalence of seasonal depression was 2.9% and the overall lifetime prevalence of depression was 26.4% |
| Probability sample of the US population; N=8098 (2836 with one or more contemporaneous depressive symptoms in the lifetime worst episode); [22] | Adult | - Timeframe: Not reported - Measures: 14 DSM-III-R symptoms of major depression - Analysis: Latent class analysis | *Identification of subgroups:*   - The 6 distinct latent classes identified were: severe typical (4%), with a high lifetime occurrence of depressive symptoms; mild typical (6%), with classical depressive symptoms; severe atypical (2%), with many depressive symptoms and symptoms characterized by appetite increase and weight gain; mild atypical (4%); intermediate (11%), with intermediate occurrence of major depression; and minimal symptoms (8%); 65% had no symptoms   *Characteristics of subgroups:*   - Severe syndrome severity (severe typical and severe atypical) was associated with a pronounced pattern of more and longer episodes, worse syndrome consequences, increased psychiatric comorbidity, more deviant personality and attitudes, and parental alcohol/drug use disorder relative to mild syndrome severity - Syndrome atypicality was associated with decreased syndrome consequences, comorbid conduct disorder and social phobia, higher interpersonal dependency and lower self-esteem, and parental alcohol/drug use disorder |
| Male-male and male-female twin pairs from population-based Virginia twin registry aged 18 to 60 years; N=2941 with at least one major depressive symptom in the prior year and comparison group of 3914 with no major depressive symptoms; [24] | Adult | - Timeframe: Not reported - Measures: 14 DSM-III-R symptoms of major depression - Analysis: Latent class analysis | *Identification of subgroups:*   - The 7 distinct latent classes were: typical (90% with major depression), characterized by symptoms of depressed mood, anhedonia, typical depressive symptoms (appetite and weight decrease and insomnia), psychomotor agitation, fatigue, difficulties with thought and concentration, worthlessness/guilt, and the highest proportion of thoughts of death or suicide; atypical (78% with major depression), characterized by depressed mood, anhedonia, atypical symptoms of appetite and weight increase, agitation, and worthlessness/guilt; non-appetitive (67% with major depression), characterized by depressed mood, anhedonia, agitation, fatigue, insomnia, difficulties with thought and concentration, worthlessness/guilt, and no appetite or weight changes; 67% met criteria for MD; 4 classes with considerably lower lifetime prevalences of MD   *Characteristics of subgroups:*   - In relation to the comparison group, the typical, atypical, and non-appetitive classes were less likely to be living with a partner, had greater lifetime trauma, more deviant personality score, greater lifetime alcohol dependence, greater SCL scores and more stressful life events - In relation to the comparison group, the typical and atypical classes had more females whereas the non-appetitive class was similar to the comparison group - Educational level was lower in the typical and non-appetitive classes relative to the comparison group - BMI was lower in the typical class and higher in the atypical class relative to the comparison group - Comparison of class assignments of monozygotic and dizygotic twin pairs showed modest evidence of familial aggregation |
| Population-based samples of community dwelling elderly adults from Amsterdam and Rotterdam; N=4051 from Amsterdam and 4603 from Rotterdam; [26] | Elderly | - Timeframe: Interviews in 1997-9 (Rotterdam) - Measures: The Geriatric Mental State-Automated Geriatric Examination In Amsterdam and the CES-D and Schedules for Clinical Assessment in Neuropsychiatry in Rotterdam - Analysis: Pre-defined subtypes | *Occurrence of subgroups:*   - Of the 523 participants who satisfied criteria for depression in Amsterdam, 42% fulfilled criteria for vascular risk; of the 333 participants who satisfied criteria for depression in Rotterdam, 50% fulfilled criteria for vascular risk   *Characteristics of subgroups:*   - Participants in the subgroup with vascular risk showed more loss of energy (both in Amsterdam and Rotterdam) and more appetite disturbance (Amererdam) - Hypothesized indicators of vascular depression, including psychomotor retardation and anhedonia, were not significantly associated with vascular risk indicators |
| ***Panic attack*** | | | |
| Community sample of the US population aged 15-54 with lifetime panic attack or disorder; N=431; [34] | Adult | - Timeframe: Interviews between 1990 and 1992 - Measures: CIDI - Analysis: Pre-defined subtypes | *Occurrence of subgroups:*   - Among those with a lifetime panic attack or disorder, the prevalence of subtypes was 51.2% for early-onset, 32.6% for agoraphobia, and 64.4% for dysthymia   *Characteristics of subgroups:*   - Early-onset panic was associated with significantly increased likelihood of bipolar disorder and substance dependence but was not distinguished from the other two subtypes by panic symptoms - Panic attack with agoraphobia was associated with significantly higher odds of several comorbid anxiety disorders, including social phobia, generalized anxiety disorder, bipolar disorder, and mania - Panic with dyspnea was more common among married females with less education and high levels of comorbid alcohol and depressive disorders |
| ***Posttraumatic stress disorder (PTSD)*** | | | |
| Male Vietnam veterans from a nationally representative sample of Vietnam theater era veterans; N=316; [31] | Adult | - Timeframe: Not reported - Measures: DES - Analysis: Subtypes based on taxometric analyses | *Identification of subgroups:*   - 76 of 316 veterans were diagnosed with current PTSD - There was evidence of a subtype of veterans characterized by a higher prevalence of symptoms of dissociation, PTSD, and dysthymia - Overall, 32% of PTSD cases belonged to the subgroup with elevated dissociative symptoms |
| ***Social phobia*** | | | |
| Representative sample of adults from Stockholm and Gotland island in Sweden; N=1202; [27] | Adult | - Timeframe: Not reported - Measures: Questions about 14 potentially phobic situations and the DIP-Q - Analysis: Hierarchical cluster analysis | *Identification of subgroups:*   - Of the 188 participants with social phobia (15.6%), the three subgroups identified were the generalized/severe (2%), non-generalized/intermediate (5.9%), and discrete/mild (7.7%) subgroups   *Characteristics of subgroups:*   - Education and social support were lowest among those in the generalized/severe subgroup relative to the other groups |
| Probability sample of the US population aged 15-54; N=8098; [28] | Adult | - Timeframe: Interviews between 1990 and 1992 - Measures: CIDI - Analysis: Latent class analysis | *Identification of subgroups:*   - Latent class analysis identified a class characterized by low endorsement probabilities, a class characterized by high probabilities of speaking fears, and a class characterized by high probabilities of multiple social fears   *Characteristics of subgroups:*   - Social phobia among those with at least one nonspeaking fear was associated with greater persistence, comorbid disorders, and impairment than social phobia among those with only speaking fears - Social phobia among those with at least one nonspeaking fear was more commonly associated with lower income and education than social phobia characterized by only speaking fears |
| Probability sample of adults from Alberta, Canada; N=1206; [29] | Adult | - Timeframe: Interviews in 1997 - Measures: Modified version of CIDI - Analysis: Subtypes based on Dice Index of Similarity | *Identification of subgroups:*   - Symptoms of fear related to speaking situations tended to be more similar to each other than other symptoms of social phobia |
| Community sample of women aged 18-24 with social phobia from Dresden, Germany; N=130; [30] | Adult | - Timeframe: Interviews in 1996 - Measures: F-DIPS - Analysis: Principal components analysis | *Identification of subgroups:*   - The number of feared social situations was distributed continuously without a clear demarcation of subtypes - Social phobics with more than four social fears were significantly more functionally impaired and had more dysfunctional attitudes than those with fewer fears - Respondents with only speaking fear had less comorbidity, subjective need for psychotherapy, functional impairment and dysfunctional attitudes than those with other social fears |

*Notes:*

CES-D=Center for Epidemiologic Studies Depression Scale

CIDI=Composite International Diagnostic Interview

DES=Dissociative Experiences Scale

DIP-Q=DSM-IV and ICD-10 Personality Disorder Questionnaire

DIS=Diagnostic Interview Schedule

DSI=Depression and Seasonality Interview

F-DIPS=Diagnostic Interview for Psychiatric Disorders-Research Version

SCL-90-R=Symptom Checklist 90-R
