# Supplementary material for: Epidemiologic heterogeneity of common mood and anxiety disorders over the lifecourse in the general population: a systematic review
Source: BMC Psychiatry. 2009 Jun 1;9:31. doi: 10.1186/1471-244X-9-31 (PMC2700109; doi:10.1186/1471-244X-9-31)
Supplement: Additional file 2 — Table two. Key studies assessing heterogeneity of trajectories of common mood and anxiety disorders. [file 1471-244X-9-31-S2.doc]

**Table 2**

Key studies assessing heterogeneity of trajectories of common mood and anxiety disorders

| **SAMPLE; N; [REF#]** | **AGE GROUP** | **METHODS** | **MAIN FINDINGS** |
| --- | --- | --- | --- |
| ***Anxiety*** | | | |
| Representative sample of kindergarten through grade 6 children from Quebec, Canada; N=1817; [51] | Child/ Adolescent/ Young adult | - Timeframe: Baseline in 1986 with yearly follow-ups until 2000 - Measures: Parents’ version of PBQ - Analysis: Semi-parametric group-based modeling | *Heterogeneity of trajectories:*   - The 4 distinct trajectories were the low group (10.2%), moderate group (39%), high group (41%), and chronic group (9.7%) |
| Boys between ages 2 and 10 from families enrolled in the WIC program in Pittsburgh; N=290; [52] | Child/ Adolescent/ Young adult | - Timeframe: Baseline interview at age 2 with follow-ups at ages 3.5, 5, 5.5, 6, 8, and 10 - Measures: Parents’ report using CBCL - Analysis: Semi-parametric group-based modeling | *Heterogeneity of trajectories:*   - The 4 distinct trajectories were the low (50.8%), low-increasing (8.8%), high-declining (32.5%), and high-increasing (7.9%) groups   *Determinants of trajectories:*   - Relative to the low trajectory group, membership in the low-increasing group was associated with children’s negative focus on delay and maternal negative control, - Relative to the low trajectory group, membership in the high-declining group was associated with higher levels of child shyness and more frequent use of passive/dependent emotion regulatory strategies - Relative to the low trajectory group, membership in the high-increasing group was associated with child shyness, maternal depression, and maternal negative control |
| General population sample of junior and high school adolescents from 12 schools in the Utrecht province of the Netherlands; N=1318; [61] | Child/ Adolescent/ Young adult | - Timeframe: 5 annual waves - Measures: SCARED - Analysis: Latent growth modeling | *Determinants of trajectories:*   - Early and middle adolescent boys experienced a decrease in symptoms of generalized anxiety disorder (GAD) over time, whereas early adolescent girls showed an increase in symptoms of GAD over time and middle adolescent girls displayed persistently higher levels of GAD symptoms - Early adolescent boys experienced the greatest decrease in symptoms of panic disorder over time - Symptoms of social phobia were higher among adolescent girls than boys, irrespective of age |
| ***Anxiety and depression*** | | | |
| Representative sample of men and women from Zurich, Switzerland who were 19-20 years old at baseline; N=4547; [54] | Adult | - Timeframe: Baseline interview in 1978 with 4 follow-ups in 1981, 1986, 1988, and 1993 - Measures: SPIKE - Analysis: Log-linear models | *Heterogeneity of trajectories:*   - Comorbid anxiety and depression was more stable over time than either anxiety or depression alone - Both anxiety and depression alone were associated with the development of comorbid anxiety and depression at subsequent interviews - Transitions from anxiety only to depression only were common, whereas transitions from depression only to anxiety only were rare |
| Members from 1946 British birth cohort (babies born in England, Scotland, or Wales during week of March 3-9, 1946); N=4627; [53] | Mixed | - Timeframe: Interviews at ages 13, 15, 36, 43, and 53 - Measures: Rutter B2 teacher questionnaire (ages 13 and 15), PSE (age 36), PSF (age 43), GHQ (age 53) - Analysis: Latent class analysis | *Heterogeneity of trajectories:*   - The 6 distinct trajectories of anxious and depressive symptoms identified were no symptoms (44.8%), repeated minor or moderate symptoms generally below threshold of mental illness (33.6%), few symptoms in adolescence but minor or moderate symptoms in adulthood (11.3%), symptoms in adolescence but not in adulthood (5.8%), few symptoms in adolescence but severe symptomatology in adulthood (2.9%), and persistent or repeated severe symptoms (1.7%)   *Determinants of trajectories:*   - Membership in more severe trajectory groups was associated with being a women and manual social class - Those with adult-onset severe symptoms or repeated severe symptoms were less likely to be married at age 43 - Those with adolescent symptoms but not symptoms in adulthood and repeated severe symptoms were least likely to have children by age 43 - More severe trajectories were associated with lower birthweight and older age at first standing and walking |
| ***Depression*** | | | |
| Early adolescents from a small city in Northwestern Quebec, Canada; N=414; [39] | Child/ Adolescent/ Young adult | - Timeframe: Baseline interview in kindergarten (age 6) with follow-up in grade 1 and each year during grades 5-8 - Measures: CDI between ages 11 and 14 - Analysis: Semi-parametric group-based modeling | *Heterogeneity of trajectories:*   - The 4 distinct trajectories were identified were the consistently low (47.7%), consistently moderate (30.3%), increasing (12.7%), and consistently high (9.3%) depression groups   *Determinants of trajectories:*   - Being in the highly depressed group was associated with female gender, a high level of family adversity, high reactivity, a poor relationship with parents, and rejection by same-sex peers (among girls with highly reactive temperament) |
| National representative sample of 12 to 25 year olds from 80 schools; N=11559; [45] | Child/ Adolescent/ Young adult | - Timeframe: Baseline interview in 1994-95 with follow-ups 1 and 5 years later - Measures: 3 items from the Depressed Affect scale of the CES-D - Analysis: Semi-parametric group-based modeling | *Heterogeneity of trajectories:*   - The 4 distinct trajectories were identified were the no depressed mood (28.7%), stable low depressed mood (59.4%), early high declining depressed mood (9.5%), and late escalating depressed mood (2.4%) groups   *Determinants of trajectories:*   - Relative to non depressed groups, membership in depressed mood trajectory groups was associated with female gender; Black/African American, Hispanic/Latino American, Pacific Islander/Asian American race/ethnicity; low socioeconomic status; greater alcohol, tobacco, or other drug use; and delinquent behavior - Relative to depressed mood groups, membership in non depressed groups was associated with a 2-parent family structure; feeling connected to parents, peers, or school; and greater self-esteem |
| Children aged 4 to 18 from the Dutch province of Zuid-Holland; N=2076; [40] | Child/ Adolescent/ Young adult | - Timeframe: Baseline interview in 1983 with follow-ups at 2 year intervals until 1991, and again in 1997 - Measures: 9 items from the Affective Problems scale of the CBCL - Analysis: Semi-parametric group-based modeling | *Heterogeneity of trajectories:*   - For males, the 6 distinct trajectories identified were the low stable (39.8%), low decreasing (39.6%), moderate increasing (14.7%), high decreasing (2.3%), high childhood peak (1.6%), and increasing high (1.9%) depression groups - For females, the 6 distinct trajectories identified were the very low increasing (13.9%), low stable (53.5%), low decreasing (19.1%), moderate stable (10.8%), adolescence onset increasing high (1.5%), and high increasing (1.2%) depression groups |
| School-based community sample of young adults from a large Western Canadian city; N=920; [59] | Child/ Adolescent/ Young adult | - Timeframe: Baseline interview in 1984-5 at age 18 with 4 follow-ups between ages 19 and 25 - Measures: 4 items from the CES-D - Analysis: Hierarchical linear modeling | *Determinants of trajectories:*   - Lower school commitment predicted higher initial levels of depressive symptoms - Increased parental education was associated with a steeper decline in young adults’ depressive symptoms - Female gender and increased conflict with parents predicted higher initial levels of depressive symptoms and faster rates of decline - Greater social support, fewer months of unemployment, and being married each predicted lower levels of depressive symptoms |
| Female and male siblings from intact families from 34 public and private schools in an 8 county area of rural Iowa; N=191 females and 185 males; [57] | Child/ Adolescent/ Young adult | - Timeframe: Baseline interview in 1989 in 7^th^ grade with 3 follow-ups at yearly intervals - Measures: SCL-90-R - Analysis: Latent growth curve modeling | *Determinants of trajectories:*   - Increasing age was associated with greater exposure to stressful life events for both boys and girls - In general, girls’ depressive symptoms increased after age 13, whereas levels remained relatively stable among boys - Exposure to a greater number of stressful life events was associated with a greater increase in depressive symptoms at subsequent waves of assessment for girls, but not for boys |
| Adolescents raised in divorced and non-divorced families in rural Iowa; N=550; [58] | Child/ Adolescent/ Young adult | - Timeframe: Various time points for 11 years (1989-99) - Measures: SCL-90-R - Analysis: Hierarchical linear modeling | *Determinants of trajectories:*   - For girls, depressive symptoms increased through middle adolescence, peaked at age 17, remained constant until age 19, and decreased thereafter; boys showed no pattern of increase in depressive symptoms - Youths whose parents divorced by age 15 had a sharper increase in depressive symptoms than their peers from non-divorced families - Stressful life events experienced after parental divorce mediated the effect of parental divorce on depressive symptoms |
| Nationally representative sample of adolescents in grades 7-12; N=10828; [60] | Child/ Adolescent/ Young adult | - Timeframe: Baseline interview in 1995 with follow-ups in 1996 and 2001-2 - Measures: 9 items from the CES-D - Analysis: Latent growth curve modeling | *Determinants of trajectories:*   - Adolescent girls started out with a higher level of depressive symptoms than boys and experienced a faster rate decline in symptoms across the transition to young adulthood - For both boys and girls, the initial level of depressive symptoms was positively associated with initial levels of smoking, binge drinking, and illicit drug use - Boys and girls with more symptoms of depression at baseline were less likely to advance to a higher category of binge drinking over time than their peers who were initially less depressed - Girls, but not boys, with more symptoms of depression at baseline had a lower odds of smoking and illicit drug use progression than those less depressed - Boys and girls who smoked, drank, or used illicit drug more frequently at baseline experienced a faster rate of decline in symptoms of depression over time than their peers who used fewer substances |
| African American adolescents at risk of high school dropout from a mid-Western city; N=579; [41] | Child/ Adolescent/ Young adult | - Timeframe: Baseline interview in 9^th^ grade with 3 yearly follow-ups - Measures: 6 items from the BSI - Analysis: Hierarchical cluster analysis | *Heterogeneity of trajectories:*   - The 4 distinct trajectories identified were consistently high, consistently low, increasing, and decreasing depressive symptoms   *Determinants of trajectories:*   - Adolescents who presented consistently high levels of depressive symptoms were more likely to be female and those with more anxiety symptoms, lower self-esteem, higher stress, and lower GPA compared with adolescents in other trajectories |
| Adolescents from 5 northern Virginia high schools; N=925; [42] | Child/ Adolescent/ Young adult | - Timeframe: Baseline interview in 2000 in 9^th^ grade with 4 follow-ups until end of 12^th^ grade - Measures: CES-D - Analysis: General growth mixture modeling | *Heterogeneity of trajectories:*   - The 3 distinct trajectories identified were consistently high (23%), moderate (44%), and low (33%)   *Determinants of trajectories:*   - For adolescents with high symptoms, baseline smoking was associated with an overall deceleration of depressive symptoms, whereas for those with moderate symptoms, baseline smoking was associated with an overall acceleration of depressive symptoms - Adolescents with high (vs. low) depressive symptoms had poorer grades, more non-sport extracurricular activity, and less physical activity at baseline |
| Sample of male students recruited in 4^th^ grade; N=206; [43] | Child/ Adolescent/ Young adult | - Timeframe: Baseline interviews in 1983-5 in 4^th^ grade with yearly follow-ups for 17 years - Measures: CDRS in late childhood and CES-D in adolescence and young adulthood - Analysis: Growth mixture modeling | *Heterogeneity of trajectories:*   - Among 15-24 years olds, the 4 distinct trajectories identified were very low (6%), moderate-decreasing (35%), high-decreasing (35%), and high-persistent (24%)   *Determinants of trajectories:*   - Negative life events and childhood depressive symptoms were positively associated with the initial level of depressive symptoms in all trajectory groups - Parents’ depressive symptoms and negative life events were associated with membership in the high-persistent trajectory relative to the other groups |
| School-based sample of adolescent students from 3 high schools in Western New York; N=975; [44] | Child/ Adolescent/ Young adult | - Timeframe: Baseline interview in 1989 with 3 follow-ups at 6 month intervals - Measures: CES-D - Analysis: Dual trajectory semi-parametric group-based modeling | *Heterogeneity of trajectories:*   - For girls, a dual-trajectory model identified 3 delinquency trajectories (41.6% rarely delinquent, 38.1% low-level delinquents, and 20.3% high-level peakers) and 3 depression trajectories (21.3% low-level depressed, 52.6% medium-level depressed, and 26.0% high-level depressed) - For boys, a dual-trajectory model identified 4 delinquency trajectories (37.9% rarely delinquent, 37.8% low-level delinquents, 17.3% medium-level delinquents, and 7% high-level peakers) and 4 depression trajectories (4.5% rarely depressed, 31.0% low-level depressed, 51.2% medium-level depressed, and 13.2% high-level depressed)   *Determinants of trajectories:*   - The level of comorbidity between delinquency and depression was higher among girls than boys - For boys, being in the rarely depressed group (vs. high-level) was associated with fewer stressful life events and childhood externalizing problems and being in the low-level depressed and medium-level depressed groups (vs. high-level) was associated with fewer childhood avoidant behaviors and childhood externalizing problems - For girls, being in the low-level depressed group (vs. high-level) was associated with fewer stressful life events, childhood externalizing problems, and childhood avoidant behaviors and being in the medium-level depressed class (vs. high-level) was associated with fewer stressful life events and childhood avoidant behaviors |
| Mothers of children born in 1991 in hospitals at 10 data collection sites across the US; N=1261; [46] | Adult | - Timeframe: Baseline interview when children were 1 month old and again at 6, 15, 24, 36, and 54 months and at 1^st^ grade (age 7) - Measures: CES-D - Analysis: Semi-parametric group-based modeling | *Heterogeneity of trajectories:*   - The 6 distinct trajectories identified were the low-stable (45.6%), moderate-stable (36.4%), intermittent (3.6%), moderate-increasing (6.2%), high-decreasing (5.6%), and high-chronic (2.5%) depression groups   *Determinants of trajectories:*   - Mothers in the low-stable depression group were more likely to be older, educated, have a higher income-to-needs ratio, stably married, and Caucasian relative to women in the other five groups |
| National probability sample of non-institutionalized adults aged 25 and older; N=3617; [64] | Adult | - Timeframe: Interviews in 1986, 1989, and 1994 - Measures: 7 items from the CES-D - Analysis: Latent growth curve modeling | *Determinants of trajectories:*   - For education, there was divergence in depression over time between college graduates and those with less than a high school diploma - For income, the difference in depression over time between high-income and low-income groups not significant and diminished in later life |
| Wife and daughter caregivers experiencing the transition from caregiving to bereavement; N=53 wives and 104 daughters; [63] | Adult | - Timeframe: 4 interviews at 18 month intervals - Measures: CES-D - Analysis: Hierarchical linear modeling | *Determinants of trajectories:*   - On average, caregivers’ depressive symptoms exhibited a curvilinear shape of change, increasing as their care recipients were closer to death and decreasing thereafter - Daughters had lower levels of depression at all times relative to wives - Caregivers of recipients with more problematic behaviors had higher depression levels and were slower to recover after recipients died compared to those who cared for elders with fewer problematic behaviors - Caregivers who experienced higher overload had a steeper increase in depressive symptoms before their care recipients died and a more rapid decline in symptoms following death - Caregivers with high income were more likely to have a downward trajectory of depressive symptoms that began before their care recipients died |
| Wife caregivers of veterans with dementia; N=1580; [47] | Adult | - Timeframe: Annual surveys between 1999 and 2002 - Measures: CES-D - Analysis: Latent trajectory class analysis | *Heterogeneity of trajectories:*   - The 4 distinct trajectories were the very low (14.0%), low (21.9%), moderate (33.9%), and high (30.3) depression groups   *Determinants of trajectories:*   - Relative to the very low group, those in the high group were responsible for their husband’s care for a longer time, reported wishing they had more help caring for their husband, and were more likely to report that their life was not very satisfying |
| Southeast Asian refugees admitted to Vancouver, Canada between 1979-1981; N=608; [62] | Adult | - Timeframe: Interviews in 1981, 1983, and 1991 - Measures: Items from the CES-D, DIS, and Senegal Health scales - Analysis: Latent growth curve modeling | *Determinants of trajectories:*   - More economically integrated refugees showed higher levels of initial symptoms of subclinical depression and greater declines in symptoms of subclinical depression over time |
| Community-based sample of bereaved caregivers; N=182; [48] | Adult | - Timeframe: Interviews at 6 month intervals including one pre-loss interview and up to 3 post-loss interviews - Measures: CES-D - Analysis: Semi-parametric group-based models | *Heterogeneity of trajectories:*   - The 3 distinct trajectories were the persistently syndromal depression (16.5%), syndromal becoming-threshold level depression (34.0%), and persistently absent depression (49.5%) groups   *Determinants of trajectories:*   - Compared to the persistently absent depression group, membership in the syndromal depression group was associated with higher behavioral burden scores before loss, fewer close relatives, worse overall health after loss, and smoking more often after loss and membership in the syndromal becoming threshold level depression group was associated with having fewer close relatives and engaging in less exercise after loss |
| Commnity sample of elderly adults age 65 and older from rural southwestern Pennsylvania; N=1260; [50] | Elderly | - Timeframe: Baseline interview in 1987 with 4 follow-ups over 12 years - Measures: Modified version of CES-D - Analysis: Semi-parametric group-based models | *Heterogeneity of trajectories:*   - 6 distinct trajectories identified included 2 asymptomatic groups, a stable low-depressed group, an emerging depressive symptoms group, a remitting depressive symptoms group, and a persisting depressive symptoms group   *Determinants of trajectories:*   - Compared to the stable-low depressed, membership in the emerging depressive symptoms group was associated with female gender and greater functional disability - Membership in the persisting compared to the remitting depressive symptom group was associated with greater prescription drug use, and having higher scores on the interpersonal/self-esteem difficulty profiles and the anhedonia/neurovegetative symptom profiles |
| Elderly adults from the general population of Baltimore; N=737; [49] | Elderly | - Timeframe: Baseline interviews in 1989-1996 with 4 follow-ups over 8 years - Measures: CES-D - Analysis: Latent state-trait models | *Heterogeneity of trajectories:*   - Variability in depressive symptoms over follow-up was mostly explained by trait-like, stable interindividual differences accompanied by state-like residual variability - The proportion of state-residual variability decreased over time |
| Probability sample of black and white adults age 65 and older from 5 counties in North Carolina; N=1972; [65] | Elderly | - Timeframe: Baseline interview in 1986 with 2 follow-ups at 3 year intervals - Measures: CES-D - Analysis: Latent growth curve modeling | *Determinants of trajectories:*   - Stress growth predicted an increase in depressive symptoms for both blacks and whites, but accounted for more variance for blacks than for whites in all but the oldest age groups |
| Probability sample of black and white adults age 65 and older from 5 counties in North Carolina; N=1972; [66] | Elderly | - Timeframe: Baseline interview in 1986 with 2 follow-ups at 3 year intervals - Measures: CES-D - Analysis: Latent growth curve modeling | *Determinants of trajectories:*   - After adjusting for age, gender, race, education, functional status, and health, there were significant associations between baseline stress and depressive symptoms and between stress growth and growth of depressive symptoms |
| Community-dwelling population of adults aged 65 and older from areas of North Carolina; N=3782; [67] | Elderly | - Timeframe: Baseline interview in 1986-87 with follow-ups in 1989-90, 1992-93, and 1996 - Measures: Modified version of CES-D - Analysis: Hierarchical linear modeling | *Determinants of trajectories:*   - The crude age trajectory of depressive symptoms in late life is positive and linear - Growth of depression was strongly cohort, rather than age, driven, with successively higher overall depression in more recent birth cohorts - Adjustment for indicators of life course (including marriage, SES, and employment status), physiological declines, and sex compositions explained both the aging and cohort effects, but particularly the cohort effects |
| ***Hyperactivity*** | | | |
| Nationally representative sample of Canadian children ages 0-23 months at baseline; N=2946; [55] | Child/ Adolescent/ Young adult | - Timeframe: Baseline in 1994 with 3 biennial follow-ups - Measures: Mothers’ report using CBCL - Analysis: Semi-parametric group-based modeling | *Heterogeneity of trajectories:*   - The 4 trajectories identified were the very low, low, moderate, and high   *Determinants of trajectories:*   - Relative to the other very low, low, and moderate groups, membership in the highly hyperactive group was associated with male gender, maternal prenatal smoking, and hostile parenting practices |
| ***Posttraumatic stress disorder (PTSD)*** | | | |
| Army personnel, including active duty, Reserves, and National Guard, who returned from the Gulf War; N=2949 at baseline; [56] | Adult | - Timeframe: Baseline in 1991 within 5 days of returning from the Gulf War with follow-ups in 1993-4 and 1997-8 - Measures: PCL - Analysis: Growth mixture modeling | *Heterogeneity of trajectories:*   - 2 distinct PTSD trajectories were found, with 57% of participants assigned to the first group, which had lower PTSD symtpomatology at baseline and little increase over time, and 43% of participants assigned to the second group, which had higher levels of initial symptoms and a significant increase over time   *Determinants of trajectories:*   - Whites and those with more education and less combat exposure were more likely to be assigned to the less symptomatic class than the more symptomatic class |

*Notes:*

BSI=Brief Symptom Inventory

CBCL=Child Behavior Checklist

CDRS=Child Depression Rating Scale

CES-D=Center for Epidemiologic Studies Depression Scale

DIS=Diagnostic Interview Schedule

GHQ=General Health Questionnaire

PBQ=Preschool Behavioral Questionnaire

PCL=PTSD Checklist

PSE=Present State Examination

PSF=Psychiatric Symptom Frequency

SCARED=Screen for Child Anxiety Related Emotional Disorders

SCL-90-R=Symptom Checklist 90-R

SPIKE=Structured Diagnostic Interview for Psychopathologic and Somatic Syndromes
